# “They Give you a bus Ticket and They Kick you Loose”: A Qualitative Analysis of Post-Release Experiences among Recently Incarcerated Women Living with HIV in Metro Vancouver, Canada

**DOI:** 10.1177/10778012231172693

**Published:** 2023-05-17

**Authors:** Margaret Erickson, Kathleen Deering, Flo Ranville, Brittany Bingham, Pam Young, Mo Korchinski, Jane Buxton, Ruth Elwood Martin, Kate Shannon, Andrea Krüsi

**Affiliations:** 1 Center for Gender and Sexual Health Equity, Vancouver, Canada; 2 Department of Medicine, 8166 University of British Columbia, Vancouver, Canada; 3 Unlocking the Gates Services Society, Vancouver, Canada; 4 School of Population and Public Health, 593205 University of British Columbia, Vancouver, Canada

**Keywords:** women, HIV, prison, incarceration, violence, homelessness, substance use

## Abstract

To explore the transition from correctional facilities to community among women living with HIV in Vancouver, Canada, we interviewed 19 recently incarcerated women and 6 service providers. Findings highlighted heightened risk of violence at release, a lack of immediate supports, challenges accessing safe housing and addictions treatment, and interruptions in HIV treatment and care. In the face of structural barriers, women blamed themselves for not being able to break the cycle of incarceration. There is a critical need for enhanced pre-release planning with a priority on housing and substance use services, alongside supports that are trauma-and violence-informed and culturally safe.

## Introduction

Involvement in the criminal legal system is inherently gendered; women are largely incarcerated for non-violent, survival-based crimes (Corsten, 2007; Kajstura, 2017) and for many women incarceration is a marker of marginalization (Currie, 2004; Herbst et al., 2016; McKendy & Ricciardelli, 2019). Globally, and in Canada, women living with HIV are increasingly overrepresented in correctional facilities relative to the general population (Dolan et al., 2016; The Correctional Investigator Canada, 2015), and HIV prevalence among incarcerated women is higher compared to incarcerated men (Correctional Service Canada, 2010; UNODC, 2019). Racialized women, including Indigenous women, are also vastly overrepresented within prison settings in many high-income countries with a history of settler colonialism (Australian Law Reform Commission, 2018; The Correctional Investigator Canada, 2018; The Sentencing Project, 2020). In Canada, Indigenous^1^ women make up 40% of all women who are incarcerated in provincial correctional facilities (remand or sentences less than two years) (Clark, 2019), despite accounting for only 4% of adult women in Canada (Arriagada, 2016). This overrepresentation must be considered within the context of historical and ongoing harms of colonial violence (Truth and Reconciliation Commission of Canada, 2015a), and structural and historical racism entrenched in the criminal legal system. Growing numbers of Indigenous women in Canadian correctional facilities highlights a continued need to act on the justice related calls to action outlined by the Truth and Reconciliation Commission (TRC) of Canada to redress the overincarceration of Indigenous women (Truth and Reconciliation Commission of Canada, 2015b).

Post-release from incarceration, women incur a multitude of unique gendered challenges (Pyper & Lalic, 2021; Substance Abuse and Mental Health Services Administration, 2020). Women involved in the criminal legal system experience higher rates of co-occurring mental health and substance use conditions compared to men, and are often the sole caregivers for children (Ventura Miller, 2021). Furthermore, research from Canada demonstrates that upon release from correctional facilities, women face significant barriers and limited supports in accessing housing, addiction treatment, employment opportunities, and healthcare (Martin et al., 2012). Although post-release programming and supports differ across settings and jurisdictions, previous research indicates gender differences in HIV health outcomes post-release among people living with HIV. This is outlined by a 2019 systematic review of research mostly from the United States, demonstrating that in the months following release from correctional facilities, cisgender (cis) women are less likely to be retained in HIV care, and experience sub-optimal HIV health outcomes - including barriers to adherence to antiretroviral therapy (ART) and viral suppression – compared to cis men (Erickson et al., 2019). Studies centering cis and transgender (trans) women living with HIV who experience incarceration across Canada highlight that experiences of incarceration intersect with various structural determinants and vulnerabilities including homelessness and unstable housing, criminalized substance use (Erickson et al., 2020; Gormley et al., 2020), and gender-based violence (Erickson et al., 2020). Among people living with HIV involved in the criminal legal system, research from the United States shows that experiencing homelessness is strongly linked to recidivism (Fu et al., 2013). Compared to men, women living with HIV are significantly more likely to experience homelessness following release from incarceration (Meyer et al., 2014) and report increased needs for social and structural supports (Williams et al., 2013). Despite this body of research, less is known about the lived experiences of recently incarcerated women living with HIV. In turn, there is a lack of innovative interventions aimed at supporting marginalized women upon release.

### Structural and Symbolic Violence as a Conceptual Framework

Structural violence, which is distinct from personal or direct violence, highlights how unequal power shapes inequities (Rhodes et al., 2012) and refers to how arrangements embedded in social structures or institutions (e.g., laws, policing practices, poverty, racism) prevent groups of people from meeting their basic needs, and ultimately causes harm (Farmer, 1996). Structural violence constitutes a significant determinant of health, and has tangible impacts on health outcomes for marginalized populations, including people living with HIV (P. E. Farmer et al., 2006). Furthermore, since structural violence is less obvious than physical violence, it is often rendered almost invisible (Scheper-Hughes & Bourgois, 2004). *Symbolic violence* is what “trails in the wake” of structural violence; making those who experience harms feel shame for their apparent “weakness” in the face of structural barriers (Scheper-Hughes & Bourgois, 2004). Pierre Bourdieu describes symbolic violence as *subtle*, whereby we often fail to recognize its very existence, let alone the way it is at the root of much violence and suffering (Eagleton & Bourdieu, 1992). In more recent iterations of structural violence, Christian and Dowler (2019), use the term *slow violence* to describe how violence can unfold gradually to the point where the “impacts of violence often come to be understood as personal and private matters, in which victims are left responsible for managing the harm” (Christian & Dowler, 2019). In other words, since harms can happen incrementally over time, blame is internalized on an individual level, instead of being allocated towards the institutions and structural systems that maintain oppression.

The concepts of structural and symbolic violence have previously been useful in framing the harms and poor health experienced by marginalized populations (Argento et al., 2011; Armstrong, 2020; Farmer, 1996; Krüsi et al., 2018; Ortiz & Jackey, 2019; Quesada et al., 2011). Drawing on more expansive definitions of violence, that centers structural and institutional forces, provides a useful framework for analyzing the intersecting gender-specific harms that are perpetuated over time by the criminal legal system. Within the context of limited consideration of the lived experiences of marginalized women who face incarceration in Canada, and the challenges women face post-release from correctional facilities, this study addresses a significant gap in knowledge by exploring the experiences of transition from correctional settings to the community among women living with HIV within a Canadian setting.

## Methods

### Research Design

This qualitative study is situated within a larger longitudinal community-based research cohort of over 350 women living with HIV in Metro Vancouver entitled the SHAWNA Project (Sexual Health and HIV/AIDS: Women's Longitudinal Needs Assessment). Recruitment for the SHAWNA cohort is ongoing, with self-identified cis and trans women invited to join the study if they are 14 years + and reside or access HIV services within Metro Vancouver. Participants complete a questionnaire at baseline and 6-month follow-up visits, alongside a sexual health component with a registered nurse, which includes HIV viral load and CD4 monitoring. To explore the lived experiences of recently incarcerated SHAWNA participants, we conducted semi-structured interviews with a subset of participants who had indicated a recent incarceration. Eligibility to participate in the qualitative interview was determined by an affirmative response (“Yes”*)* to the following question within the cohort questionnaire: “In the last 6 months, have you been in detention, prison or jail overnight or longer for any reason at all?”. All participants who indicated a recent (last 6-months) incarceration during any baseline or follow-up questionnaire within the last 5 years (at the time of study recruitment in 2017) were contacted and invited to take part in this qualitative study. Of the 30 SHAWNA participants who were identified as eligible to participate, 22 participants were booked in for an in-depth interview. Among the remaining 8 eligible participants, 2 had moved away, 5 could not be reached (including due to missing contact information), and 1 had passed away. Three participants missed their appointment (despite multiple attempts at re-booked appointments), and as such 19 participants took part in the semi-structured interviews.

To complement interviews with SHAWNA participants, we also conducted interviews with key service providers who work directly with women living with HIV experiencing incarceration in British Columbia (BC). With input from SHAWNA Project partners and facilitated through long-standing community relationships with HIV service organizations and health care providers, service providers were purposely sampled to include those who work with women along incarceration trajectories (e.g., from community-based and HIV support organizations and women-centered HIV clinics). Snowball sampling served to add additional service providers. Participants were contacted via email, or by phone. Drawing on narratives from both women with lived experience of HIV and incarceration and service providers allowed for triangulation of findings (Creswell & Poth, 2017).

### Data Collection

Between May 2017 and February 2018 three experienced interviewers including one Indigenous woman living with HIV with a history of incarceration, conducted semi-structured interviews with 19 SHAWNA participants who had been recently incarcerated. Interviews took place at one of two research office locations within Vancouver, on the traditional unceded territories of the xʷməθkwəy̓əm (Musqueam), Skwxwú7mesh (Squamish), and Səl̓ílwətaʔ/Selilwitulh (Tsleil-Waututh) Nations. All participants provided informed written consent. Interviews were audio recorded and lasted between 35 and 120 min. Participants were remunerated with a CAD $30 honorarium for their time and expertise and provided with bus fare for travel if needed.

The semi-structured interview guide was developed, revised, and piloted in collaboration with the SHAWNA peer researchers and community partners. Participant demographics were collected using a brief survey, which included questions assessing personal demographics, and specifics relating to incarceration (e.g., number of lifetime and recent arrests, information regarding the most recent incarceration). During the semi-structured interview, participants answered a series of questions exploring experiences navigating incarceration. This included a focus on the time leading up to release from custody and pre-release planning, as well as questions surrounding post-release trajectories, including experiences in the immediate hours and weeks following release, and challenges and facilitators accessing services, supports, and HIV care.

Between February and May 2021, two team members conducted interviews with 6 service providers. The interviews explored experiences providing care and supports for women living with HIV, including but not limited to logistics and unique challenges navigating care within the criminal legal system, barriers and facilitators surrounding transitional supports post-release from incarceration, and overall impacts of incarceration for this population. Service provider interviews were conducted over the phone or via Zoom to facilitate physical distancing during the COVID-19 pandemic. Service providers at peer-led organizations who identified as people with lived experience of incarceration (n = 2) were compensated CAD $30 for their time and expertise. The study holds approval by the Providence Healthcare/University of British Columbia Research Ethics Board—H14-01073.

### Data Analysis

Audio recordings were transcribed verbatim, de-identified, and checked for accuracy. The transcripts of the 19 semi-structured interviews with SHAWNA participants were coded in an iterative process using deductive and inductive approaches involving the use of a priori and emergent categories (Bradley et al., 2007). The coding framework was developed through discussions and input from the SHAWNA peer research team (women living with HIV with incarceration experience). The framework comprised of categories derived from the interview guides (e.g., pre-release concerns, HIV-care post-release, housing post-release etc.) and then expanded to include emic categories emerging from the interview data (e.g., feelings of anxieties pre-release, urges to use drugs immediately after release, relationships with HIV-specific support workers post-release). To advance beyond thematic description, we drew on concepts of structural and symbolic violence (Eagleton & Bourdieu, 1992; Rhodes et al., 2012) – as described above – to give focus to how structural barriers shaped and perpetuated incarceration trajectories. The transcripts of the interviews with the 6 service providers were coded using the same process, with a deductive approach drawing on themes and data from the 19 interviews with SHAWNA participants, and an inductive approach based on additional categories that emerged from the interview data (Bradley et al., 2007). The data was coded by the lead author with substantial oversight and guidance from the senior author and SHAWNA peer research team (described above). We used NVivo 12 software for coding and applied pseudonyms to protect the confidentiality of participants.

Drawing on participatory-action research and to increase analytical rigour (Creswell & Poth, 2017), we convened a small advisory working group (n = 5) of women living with HIV with lived experience of incarceration (including women who had participated in the interviews for this study) to discuss and conceptualize the main themes and findings. During two sessions, themes and findings from the analysis were discussed amongst the working group in relation to their own experiences and knowledge. These sessions took place over Zoom (April/May 2021) and were co-facilitated by SHAWNA project staff with lived experience of HIV.

## Results

Table 1 depicts demographic characteristics of the 19 SHAWNA participants. At the time of the interview, the majority (n = 17) identified as cis women and two Indigenous participants identified as Two-Spirit.^2^ Both Two-Spirit participants only shared experiences of short-term stays at local city jails (i.e., commonly overnight or weekend stays), therefore the experiences presented in this analysis are from cis women who experienced longer episodes of incarceration (most commonly at provincial-level correctional facilities). Participants mainly spoke to experiences of incarceration between 2011 and 2017 within Metro Vancouver, with the exception of a few participants who also discussed experiences of incarceration in Canadian provinces outside of BC. Experiences of sustained cycles of criminalization were common among participants; over a 5-year period, 6 participants had been arrested between 6 and 10 times, while the remaining 13 participants reported being arrested up to 5 times in the same period. Twelve of the 19 participants identified as Indigenous, reflective of the disproportionate criminalization and incarceration of Indigenous people in Canada (Correctional Service Canada, 2010).

**Table 1.** Demographic characteristics of recently incarcerated women living with HIV

Total number	19 (100%)
Median age (range)	45.5 (36–55)
Gender (n, % of total)	
*Cisgender woman*	17 (89.5%)
*Two-Spirit*	2 (10.5%)
Indigenous	12 (63.2%)
White	7 (36.8%)
Average age at first incarceration (range)	21.7 (13–45)
Number of arrests since 2011 (n, % of total)	
*1–5 arrests*	13 (68.4%)
*6–10 arrests*	6 (31.6%)
Most recent incarceration	
*Held at city cells only*	5 (26%)
*Served time in a provincial correctional facility*	14 (74%)
*Average time served in months (n* *=* *14) (range)*	3.25 (.25- 24)
Daily criminalized substance use at the time of the interview	11 (57.9%)

The service providers were individuals who had current or former roles working with women, and women living with HIV specifically, during and post-release from incarceration in Metro Vancouver, including HIV-prison outreach workers from community service organizations, HIV physicians and nurses from women specific HIV clinical care settings, and peer-support workers from peer-led organizations.

### Pre-Release

#### Planning, Supports, and Pre-Release Anxieties

Most participants reported that they received little to no pre-release planning (i.e., support with needs at release including housing, addiction treatment programs, transportation, employment opportunities, etc.) from corrections staff or outside support organizations. Service providers noted that this was not uncommon, citing limited corrections-based resources allocated to ensuring adequate transitional support, along with short stays in custody (often just a few weeks), and challenges around release planning due to difficulties predicting release dates: *“*All of a sudden, the lawyer would advocate to get somebody out earlier. I often found the women didn’t know themselves when they were getting out for sure - it just made it so much more complicated to actually come up with a plan” [service provider #4]. In addition to limited pre-release planning many participants discussed strong feelings of anxiety and fear in the time leading up to release. Lucy detailed how being released into the community with no supports, after months of being institutionalized made her feel overwhelmed and brought up suicidal ideation: “When they let me out after being inside for almost six months, I was fucked up… I wanted to walk in front of a semi [truck]…I never want to feel like that again” [Lucy, white]. To cope with these intense feelings, Lucy returned to criminalized substance use the day of her release.

For many participants the anxiety and fear leading up to release was linked to struggles with criminalized substance use. Marlene, an Indigenous participant, recounted: “I remember praying…asking the Creator to give me strength to stay away from the drugs and to you know, do something better with my life…I didn’t wanna go back to [my situation prior to incarceration]”. Similarly, Yvonne described that her “heart was racing” prior to release, she expressed that she “wanted to stay clean” but received very limited support. When asked to describe what happened immediately post-release, she replied: “I went out and got high” [Yvonne, white]. For some participants, narratives also highlighted that drug use served as a form of coping with trauma. For example, Diane discussed her pre-release anxiety, and how using substances helped her cope with the pain of her child not being in her care: “I get the butterflies [pre-release]…my mind was focused on getting high again…I was anxious. But part of me wanted to get clean as well…I had a hard time…I just wanted to get my baby back [her child]. But I knew that wasn’t gonna happen. So, I chose to get high instead” [Diane, Indigenous]. Diane's narrative speaks to criminalized substance use as a coping method for the trauma and grief associated with child apprehension (i.e., a child being removed from a parent/guardian's care by social services) (Kenny, 2018; Thumath et al., 2020). Diane's experience also highlights the ongoing impacts of colonialism and structural racism by way of the cyclical nature of surveillance and institutionalization for generations of Indigenous women and their children^3^ (Chartrand, 2019; Mussell, 2020). Service providers stressed the need for enhanced pre-release supports for women during this sensitive time in order to address the often complex anxieties and fears: “[We need to] work with people before they get released…talk about why [they’re] having these mental obsession [with drugs], what is the fear…what are [they] nervous about [post-release]… [we need] to address these issues. We’d have way better luck [with post-release success]” [service provider #5]. However, in the absence of adequate pre-release planning and supports, many participants experienced substantial challenges in the immediate hours following release from correctional facilities.

### Challenges Immediately After Release

#### Transportation, Clothing, Stigma, and Vulnerability to Violence

For many participants, transportation back to the community constituted a significant struggle and period of vulnerability. Participants’ narratives highlighted that those who did not have someone to pick them up upon release from the correctional facility – which was often located in an unfamiliar city a significant distance from their home community – were left to navigate a multi-jurisdictional public transit system. As described by one participant: *“*They give you a bus ticket and they kick you loose…you have to find your way back. I had no idea where the hell I was” [Beth, white].

Char, an Indigenous woman who reported receiving no pre-release planning support despite being incarcerated for 11 months, described: “They just said, ‘see ya’. [I] even left in a monkey suit [prison clothes].” Being released from custody wearing prison issued clothing is common for various reasons, including if the clothes someone was arrested in no longer fit, if they are not suitable for the weather, or if individuals are released directly from court without access to their personal belongings. A peer service provider with incarceration experience noted: “It's sad coming out with jail clothes. Like, how do you survive, especially when you’re trying to change your life, right?” [service provider #6]. In addition to this experience being humiliating and demoralizing, prison issued clothing increased public visibility, outing women as recently released from corrections, violating their dignity, and re-enforcing the stigma surrounding people involved in the criminal legal system.

To avoid public visibility, the need to access a change of clothes immediately post-release put some participants at risk of re-arrest, as explained by Char: “The first thing I did [when I got out] is I went and stole some clothes. Cause I was in one of those prison uniforms”. Others had to engage in informal economy to raise funds for basic needs and were exposed to the potential of violence due to their heightened vulnerability and economic need. This was described by Beth, who had no means of immediate financial support post-release: “I stopped in [Metro Vancouver suburb] and I pulled a trick [sex work]. I didn’t know what else to do [for money]… [I was] in jail clothes, it was ridiculous”*.* Similarly, Rita highlighted how, aware of the immediate and often desperate financial needs of recently released women, some taxi drivers - meant to facilitate transport from the correctional facility to the local bus stop - tried to take advantage of the economic situation of recently incarcerated women: “[the cab drivers] would be asking the girls before they even got to the bus stop, if they wanted to pull a date [provide sexual services for a fee]” [Rita, white]. This was also reiterated by service providers who highlighted that despite plans to refrain from using drugs upon release, the absence of supports in the immediate post-release hours contributed to women's vulnerability:

[Cab drivers know] these women are desperate. So even if somebody was hanging on by their fingernails… and [had] no intentions of getting [high], you have a cab driver right off the bat saying ‘hey… give me a blow job and I’ll give you thirty dollars so you can go get high and I’ll take you to a drug house’… there goes anything in that woman's [plan]…all of a sudden poof. [service provider #5]

These narratives highlight the structural violence of releasing women from correctional facilities without supports and illustrates the relationship between a critical lack of transitional supports and increased risk of harms. One service provider added: “The correctional system did not ask…or seem like they cared where the women [would end up]. It was like: released date, door closed, done” [service provider #4].

### Lack of Supports in the Post-Release Period

#### Housing

The lack of structural supports post-release was evident among most participants when it came to housing options. Although having a safe place to stay was one of the most pressing needs post-release, participants described significant challenges and barriers to safe and accessible housing.

*Homelessness, Gender-Based Violence, and Health*. With limited to no options for safe and supportive housing, many participants ended up homeless after release from corrections. Others opted to move in with their partners or boyfriends, however this was not always the best or safest option, as described by Anna who was staying in her partner's low-income, single room occupancy building: “I didn’t like it. It was small and there [were] cockroaches… [we] had to share two washrooms with like, twenty other men…It was horrible but I had to stand it. I wanted to go stay at a women's shelter but [my boyfriend] didn’t want me to go stay somewhere else” [Anna, Indigenous].

Anna's narrative alluded to her inability to seek alternative housing arrangements as a result of the control imposed by her partner, further highlighting how the structural violence of no safe housing supports after release from correctional settings can put women at increased risk of gender-based violence. Several participants spoke to incidences of experiencing violence from abusive partners which led to involvement from police, and often times the women themselves being arrested and charged (e.g., for assault or for breaching conditions etc.). Service providers spoke to this as a common phenomenon and echoed the importance of access to safe housing post-release: “Housing [makes] a huge difference because [then they’re] not staying at [their] abusive boyfriend's house where the cops are gonna get called all the time. [They] actually have some safety and some security in [their] own space, and it makes a really big difference” [service provider #1].

Beth described the exhausting and challenging experience of being homeless post-incarceration, and how this presented a significant barrier to well-being and perpetuated a cycle of incarceration: “[After release] I was living on the streets…you lose everything, you start all over again every time you go in [to prison]…and of course if I’m on the street, I’m gonna be using [drugs] just to survive… then anytime the police see you… they just take you in [to jail] overnight, so it's super stressful… it's just a circle of accomplishing nothing” [Beth, white].

Experiences of continued HIV care during the transition from corrections to community varied among participants. Some women were provided with ARVs upon release from correctional facilities and others made follow up appointments with their HIV care providers in the days and weeks post-release. Others noted not receiving any ARVs or supports with regards to continuity of care. For some participants, structural violence by way of lack of housing supports and stability during this period was linked to challenges with adherence, as further described by Beth: “You’re supposed to be in a stable environment when you’re on your antivirals, right?…When you’re constantly having your stuff stolen, and you got nowhere to stay then it's impossible” [Beth, white]. Service providers echoed the importance of stable housing for maintaining adherence post-release: “more often than not women weren’t calling us to get their meds refilled because they’re homeless and [HIV care is] not their priority at that time - their safety and security is - so of course health gets prioritized way lower” [service provider #4].

*Housing Instability, Criminalized Substance use, and Unregulated Recovery Houses*. With limited options for housing and/or addiction treatment supports post-release from incarceration, some participants ended up in “recovery houses”. These homes are often co-ed and can be largely unregulated as described by one service provider: “[After getting released from court] I drive her to this recovery house – we knock on the door and some dude answers, no shirt, holding a crack pipe, and he's like ‘oh I’m sure we can rustle up a mattress somewhere’” [service provider #1].

One participant, Rita, recounted that despite vocalizing her concerns, she was court-ordered to a recovery house in an area where she had a previous history of engaging in drug use. Pushing back against the symbolic violence of being expected to stay abstinent from criminalized drugs under inadequate, unsafe, and challenging circumstances, Rita noted that she was “being completely set up for failure” by having to live in a neighborhood with a significant open drug scene. Another participant, an Indigenous woman named Susan, detailed her experience with a post-release recovery house. She noted: “There was nothing safe about it at all”, explaining that the majority of residents were men whom she described as “really creepy”, and who were openly using drugs in the house. A staff member, whom she felt was not qualified to dispense HIV medications, outed Susan's HIV status without her consent. She detailed: “He yelled up at me, he goes, ‘sorry to hear about your HIV, [Susan]!’…And we were at supper. [The residents] were having dinner.” These experiences made Susan feel highly unsafe and she opted to leave the recovery house. However, since she was court-ordered to reside there, the housing manager continued to collect rent, leaving her without alternative housing other than to couch surf – which she described as dangerous. Susan's narrative is an example of the harms perpetuated by the structural violence of unsafe release conditions imposed by the courts that can expose marginalized women to breaches of confidentiality, highly unsafe housing situations, and homelessness.

Finally, for some participants, being incarcerated had provided space to consider addiction treatment programs, which, as outlined above, might also offer temporary housing solutions. However, a lack of available safe addictions treatment options in BC coupled with significant waitlists constituted a barrier, as explained by Marlene: “I tried to stay clean [while waiting to get into treatment], but the addiction took over and I started using again…then I started getting down on myself about it and going back to my old lifestyle. [I pressed] the fuck it button.” [Marlene, Indigenous]

In the absence of structural supports during the post-release period, many participants ended up blaming themselves for relapsing, turning back to their old lifestyle or being re-incarcerated, highlighting the symbolic violence (Eagleton & Bourdieu, 1992) resulting from the criminal legal system. A common theme stemming from participant's stories was the significantly disruptive nature of short-term incarceration, which, coupled with a lack of post-release supports, maintained the cycle in and out of the system, often referred to as the “revolving door”.

#### Designated Post-Release Supports

Access to designated programs during the transition back to community was rare among participants. However, those who were connected to support organizations recounted how non-judgmental and supportive relationships with service providers was key to trajectories of resiliency. Two participants recounted experiences with the same support worker from an HIV-specific prison-based outreach program who had connected with them during their incarceration and supported them with multiple aspects of re-integration. Lucy described her experience, illustrating the significance of this relationship during and post-incarceration: “She's my rock. She's the whole reason I got through [prison]… She's in my life now, right? We go for walks, we go have breakfast or lunch sometimes… without her, man I probably wouldn’t be here. I probably would have offed myself a long time ago, or who knows what” [Lucy, white]. The other participant who connected with this support worker described: “She took me herself [to appointments]… it made a big difference, like a big sister… and that's why she's my hero… am I worth it? She showed me that…she's just wonderful…we need her” [Susan, Indigenous].

Although significant in highlighting the need for trusting relationships and supports for women involved in the criminal legal system, these narratives also highlight the symbolic (Scheper-Hughes & Bourgois, 2004) or *slow* violence (Christian & Dowler, 2019) of the absence of supports for women after release from correctional settings. Participants’ narratives about the difference a support worker can make even in the context of limited available resources underscored how the absence of supports after release from corrections are often internalized on an individual level, rather than attributed to harms perpetuated over-time on a structural level. One peer service provider with lived experience of incarceration echoed this sentiment based on their own experiences:

There's more to [being released] than just walking out [of prison] and making sure you have a bed…a treatment center…a safe place. There's so much more that goes on […]. We don’t think we’re good enough…we’ve been broken down as people for so long… Mainstream society doesn’t take care [of us] because they think we’re in this position because you know, it was our choice [service provider #5].

In the wake of complex emotions and stigma during this critical transition period, participants who did not have access to support organizations noted how significant it would have been during the transition to be connected to a designated support worker, peer (i.e., someone with lived experience of incarceration), or healthcare worker to help them through this time. As one peer service provider explained: “there's just so much judgement and stigma [post-release]… it's really hard, you know? If you can have that person who believes in you it makes all the difference in the world” [service provider #6].

## Discussion

The findings of this study highlight intersecting structural barriers experienced by women living with HIV upon release from correctional facilities including limited pre-release planning, a lack of immediate supports at release, challenges accessing safe housing and addictions treatment, and interruptions in HIV treatment and care. The structural violence perpetuated due to a lack of supports post-release perpetuated symbolic violence leading many women to blame themselves for not being able to escape the cycle of incarceration and criminalization. Conceptualizing the experiences of marginalized women involved with the criminal legal system as a form of structural and symbolic violence foregrounds the connections between structural inadequacies and the intersecting harms and vulnerabilities that are perpetuated by the criminal legal system. As summarized succinctly by anti-oppression, abolition and liberation educator Rania El Mugammar, “the guilt belongs to the system” (El Muggabar, 2021).

Although women involved in the criminal legal system face many intersecting forms of marginalization and subsequent challenges – highlighted by this study – for Indigenous women specifically, the unjust cycle of incarceration must also be understood in the context of colonial violence that perpetuates structural inequities, and ignores the historical systemic injustices rooted in colonialism (National Inquiry into Missing and Murdered Indigenous Women and Girls, 2019). This study supports calls to action for meaningful commitments to address the continued colonial violence perpetuated against Indigenous women (National Inquiry into Missing and Murdered Indigenous Women and Girls, 2019), and eliminate the over-representation of Indigenous people among incarcerated populations (Truth and Reconciliation Commission of Canada, 2015b).

Our findings add to research that underscores the lack of gender-specific supports and programming for women leaving correctional settings (Janssen et al., 2017; Salem et al., 2021; Ventura Miller, 2021). Consistent with other women who face incarceration across Canada (Cram & Farrell MacDonald, 2019), the majority of participants in our study experienced significant struggles related to criminalized substance use. The close relationship between criminalized substance use and incarceration among women is well-documented (Gobeil et al., 2016; Johnson et al., 2012; Strathdee et al., 2015); and our study adds to research that illuminates the complex substance use-related anxieties and risks that exist for women leaving correctional facilities (Elwood Martin et al., 2019; McLeod et al., 2020). This is of critical concern given the contaminated illicit drug supply that has fueled an explosive and deadly overdose epidemic in BC (British Columbia Coroners Service, 2022) and across North America (Centers for Disease Control and Prevention, 2021).^4^ Previous research has identified release from incarceration as a time of heightened risk for overdose and death (Binswanger et al., 2007; Gan et al., 2021). Reports from the First Nations Health Authority in BC indicates that in 2020, First Nations women died from overdose at a rate almost 10 times that of non-Indigenous women (First Nations Health Authority, 2021), highlighting the devastating toll of drug toxicity on the lives of Indigenous women and their communities. This study highlights how an absence of accessible substance use supports after release from incarceration can leave women feeling defeated and blaming themselves for relapsing. A lack of comprehensive supports surrounding criminalized substance use is a structural failure that can be fatal, and as such this work underscores and echoes the need for comprehensive substance use supports, specifically for women involved in the criminal legal system (Salem et al., 2021).

Participants in our study recounted major barriers to accessing safe and secure housing following incarceration. These findings align with research from a study of women released from provincial corrections in BC, whereby at release 85% of participants were either homeless or unstably housed (McLeod et al., 2020). For study participants who were placed in “recovery houses” upon release, their narratives echo previous accounts illustrating how these spaces are unregulated and often unsafe for women (Homles, 2019; Woodward, 2012). As highlighted by the National Inquiry for Missing and Murdered Indigenous Women and Girls, the current transitional housing system fails to provide comprehensive and safe housing supports and instead places women at risk of further harms while maintaining cycles of incarceration (National Inquiry into Missing and Murdered Indigenous Women and Girls, 2019). Participants’ experiences of being court-ordered to these types of unregulated and unsafe recovery houses again highlights the structural violence of harmful and under-resourced policies. The absence of structural supports in the form of safe housing was also linked to interruptions in HIV treatment and care for some participants, further highlighting how inadequate post-release housing shapes health outcomes. Research has consistently shown that homelessness and unstable living situations constitute significant barriers to continued HIV treatment during the transition from incarceration to community (Meyer et al., 2011; Nunn et al., 2010), while stressing the importance of supportive transitional housing for women (trans inclusive) in promoting adherence to ART post-release from incarceration (Ghose et al., 2019). Culturally safe models of housing supports and interventions are also needed (Bingham, 2020; Christensen, 2013; Thistle & Smylie, 2020) to support the safety and specific needs of Indigenous women. This includes supports for Indigenous programming in transitional housing, as outlined by the TRC (Truth and Reconciliation Commission of Canada, 2015b). Studies also point to an intersection between housing instability, criminalized substance use and decreased adherence to ART (Friedman et al., 2009; Haley et al., 2014), which is especially pronounced for women living with HIV who experience incarceration (Chitsaz et al., 2013; Nunn et al., 2010). The results of our study further underline calls for comprehensive pre-release planning and post-release services that encompass supports for both HIV care as well as criminalized substance use (Swan, 2015) and re-iterate the immediate need for safe and women-specific housing options – including housing specific to addictions treatment – as critical in supporting HIV health and general well-being during re-integration.

Our findings further elucidate the intersections between gender-based violence, housing instability, and the cyclical nature of incarceration among marginalized women. Many participants who experienced housing instability post-release expressed how this increased visibility to police and thus risk of re-arrest. Similarly, service providers stressed the importance of housing options in providing women with their own space away from abusive relationships. Experiences of intimate partner violence perpetuated against women involved in the criminal legal system are exceedingly high (McMillan et al., 2021). Research demonstrates increasing and often inappropriate charges against women in instances where police are called to address intimate partner violence (Grace, 2019), which further perpetuates the cycle of incarceration. This reality also disproportionally impacts Indigenous women who are exposed to higher rates of violence and then criminalized for protecting themselves or their children (National Inquiry into Missing and Murdered Indigenous Women and Girls, 2019). As such, this research underscores the TRC justice related calls to action for additional culturally relevant services during incarceration, including supports that address experiences of violence and abuse, along with added programming for Indigenous people in transitional housing, and those being supervised in the community by corrections (Truth and Reconciliation Commission of Canada, 2015b). Given the unique needs of women upon release from correctional facilities, including heightened vulnerabilities and exposure to gender-based violence, it is essential that all transitional supports – including housing and substance use services – be tailored appropriately to include gender-specific options (World Health Organization, 2011) rooted in trauma and violence-informed practice (Equip Health Care, 2021).

Finally, supports that focus on connecting women to services – including peer-based programs – that build and maintain trusting relationships, remain crucial in the post-release period. Connections through community and peer-based relationships and initiatives during the transition from incarceration have shown to be instrumental (Elwood Martin et al., 2019; Heidemann et al., 2014; McLeod et al., 2020). Research with women living with HIV in other settings demonstrates how being connected to a designated service provider during this transitional period can increase feelings of support and self-confidence (Fuller et al., 2019; Koester et al., 2014; Nunn et al., 2010), and in some cases directly impact increased adherence to ART (Nunn et al., 2010). However, despite growing rates of incarceration among women living with HIV, the prison-based outreach program specifically referenced by participants in our study has since lost funding and has been terminated. The loss of funding has been consistent across many other HIV-specific community-based organizations and supports within Canada, including supports specific to women living with HIV (Easton, 2016). This has left a gap in supports for marginalized women involved in the criminal legal system. Though some participants recounted significant and positive experiences accessing HIV-specific prison outreach programs post-release, there was an overall lack of accessible designated support services for participants during the transition, which impacted optimal HIV health. Our findings support a critical need for the re-allocation of funding to support women living with HIV during the transition period, with an emphasis on culturally safe, peer-led programming.

### Limitations

Our findings mainly speak to the experiences of women living in an urban setting who were incarcerated in the main provincial facility for women in BC. Further research is needed to elucidate the unique experiences of women who leave correctional settings to return to rural communities, as well as those from other jurisdictions. The two Two-Spirit participants who were interviewed for this study had been held overnight in city jail cells and did not speak to experiences transitioning back into the community after being incarcerated for longer periods of time. Since correctional facilities operate on a gender binary, and often do not support gender affirming practices for trans people (Van Hout & Crowley, 2021), there is limited research regarding the experiences of incarceration, including the impact on HIV care and transitional supports for trans, Two-Spirit, and non-binary people living with HIV. Research exploring their unique experiences and needs upon release is urgently needed. Our sample drew from the SHAWNA cohort, and reports of recent incarceration in this sample were limited to white and Indigenous participants. Further research that centres the experiences of Black and other racialized women living with HIV in Canada remains pressing. A note on a limitation of the semi-structured interview guide: although many women who experience incarceration are mothers whose children are no longer in their care (Corsten, 2007), we specifically chose not to include questions in the interview guide that asked about children/reunification with children post-release from incarceration given the potential of bringing up trauma during the interview. Despite this, several participants recounted painful experiences of navigating the histories and relationships with their children. Finally, it is important to note that since the interviews for this study were conducted, changes have been made at an organizational level within BC Corrections. In October 2017, the healthcare delivery in BC provincial correctional facilities was assumed by the Provincial Health Services Authority; a focus has been increased mental health and substance use supports among people who are incarcerated, including the implementation of community transition teams aimed at improving linkages to care and other supports following release from custody (BC Mental Health and Substance Use Services, 2019). Although a formal gender-based evaluation of the impact of the transition teams will be important, these developments will hopefully lend to enhanced transitional supports and improved outcomes for marginalized women involved in the criminal legal system.

### Conclusion

In conclusion, this study elucidates the intersecting structural barriers experienced by women living with HIV transitioning from correctional facilities to the community, which perpetuated structural and symbolic violence and highlighted how involvement in the criminal legal system is sustained among marginalized women. This study further reiterates calls for action to redress the overincarceration of Indigenous women in Canada. To improve HIV health outcomes and overall well-being for all women living with HIV following incarceration, there is a critical need for increased transitional supports from correctional facilities to community. Enhanced pre-release planning with a priority on safe housing and addictions treatment options remain critical to improving HIV care and addressing cycles of criminalization and incarceration. To meet the unique needs of women involved in the criminal legal system, interventions and programming should be gender-specific and rooted in trauma-and violence-informed practice and cultural safety. Whenever possible options for peer-supports should be prioritized.

## Notes

1. In Canada, this term refers to First Nations, Métis, and Inuit people.

2. Two-Spirit is an identity among people Indigenous to Turtle Island (otherwise known as North America) who identify as having both a masculine and a feminine spirit, and may be used to describe any or all of sexual, gender and/or spiritual identity; however, this depends on the individual and context (Pruden, 2019).

3. The final report of the National Inquiry into Missing and Murdered Indigenous Women and Girls points to the fact that the majority (64%) of Indigenous women who are incarcerated are single mothers, which often results in the institutionalization of their children at an early age by way of the foster care system (National Inquiry into Missing and Murdered Indigenous Women and Girls, 2019).

4. Since the time of interviews (2017/2018), two participants have passed away from overdose. We honour the lives of these women, and are immensely grateful that they shared their stories of lived experience with our team. Their passing reflects the continued devastating impacts of the overdose crisis on the lives of marginalized women and their communities. These deaths also only represent a fraction of SHAWNA participants who have lost their lives to overdose during the course of the study.
